# Insights from Parents about Caring for a Child with Birth Defects

**DOI:** 10.3390/ijerph10083465

**Published:** 2013-08-07

**Authors:** Jodi Lemacks, Kristin Fowles, Amanda Mateus, Kayte Thomas

**Affiliations:** 1 NBDPN Parent Advisory Group, 8150 N. Central Expwy., M2248, Dallas, TX 75206, USA; 2 NBDPN Parent Advisory Group, 11 Michael Townsend Court, Newark, DE 19702, USA; E-Mail: Kristin.fowles@gmail.com; 3 NBDPN Parent Advisory Group, 601 N 300 W, Spanish Fork, UT 84660, USA; E-Mail: amandalmateus@gmail.com; 4 NBDPN Parent Advisory Group, 3122 Wilder St., Raleigh, NC 27607, USA; E-Mail: indigoeyes@ymail.com

**Keywords:** birth defects, parents, quality of life, communication, transition of care, prevention, advocacy, awareness, NBDPN

## Abstract

Birth defects affect 1 in 33 babies. Having a child with a birth defect impacts the whole family. Parents of children who have birth defects face unique challenges and desire to make life better for their kids. They also want to help to prevent birth defects in the future. Some of the challenges parents face involve communication with healthcare professionals, quality of life issues, creating awareness and advocating for research and funding, finding resources and support, and helping teens transition to appropriate, specialized adult care. This paper addresses these issues and their sub-issues, provides examples, and makes suggestions for improvement and research.

## 1. Introduction

Birth defects affect us all, but particularly families with children who have birth defects. With 1 in 33 babies currently born with a birth defect, and with birth defects being a leading cause of morbidity and mortality in children, there are issues that need to be addressed to help all families be healthier and happier [1]. First, parents need others to be aware of the impact of birth defects and how they affect quality of life within families and in the children. Second, parents would like better communication from and with healthcare professionals so that they are better able to care for their child. Third, prevention of birth defects is not only essential for the health of future generations, but also to prevent some of the co-morbidities that come with the birth defects. Parents can be extremely helpful in awareness and funding for prevention, but they need to be connected with a common cause. Finally, every parent wants their child to reach adulthood and then move on to old age, but because many children, until recently, did not survive to adulthood, the resources to help parents and teens make this transition are often not available. Parents need these resources to ensure their children get adequate, and sometimes life-saving, care throughout their life cycle. As parents of children with birth defects, the purpose of this article is to share our thoughts and experiences with other parents, researchers and health care providers. Our hope is to address four main themes common with the majority, and even perhaps all of birth defects, issues within these themes, and include many suggestions as to how to best care for these patients and their families.

## 2. Quality of Life

One of the most devastating, life-changing events for parents is finding out their child has a birth defect. What they do with this news depends on many factors: it depends on the knowledge, attitude, and style of communication of the provider giving the diagnosis; it depends on the information they are given after diagnosis and whether they are connected to the appropriate services and support groups; and, it is affected by personal beliefs, culture, education level, and support available to the parents. 

### 2.1. During Pregnancy (Prenatal)

The first thing most parents want to know about their unborn baby is whether the baby is healthy. When parents learn about a baby’s birth defect in the prenatal period, they are often devastated. They may become so preoccupied with their baby’s medical condition and medical visits that they cannot enjoy their pregnancy. Some mothers even forgo baby showers and other celebrations because they feel somehow undeserving or fearful of the post-natal outcomes. For these parents, taking the time to relax and feel “normal” can help to relieve stress, but it is often very difficult. They wonder what their life will be like with a baby who has a birth defect—many times a birth defect they have never heard of or seen. Images of their baby sick and dying, which may be a reality, haunt the rest of the pregnancy. Parents are often faced with many tough decisions during this time, and they need reliable and accurate information. Compassion, caring and connecting others who have traveled a similar path may also help parents immensely during this time [2,3].

### 2.2. Post-Diagnosis

After a child is diagnosed with a birth defect, parents often go through stages of grief similar to those they would have if they had lost the child. While that may seem odd to many, the parents actually did lose a child—the “normal,” healthy child that they were expecting. Before diagnosis, parents had a vision of life with their child that changes drastically post-diagnosis. It is normal to experience the typical stages of grief as parents mourn the loss of what they expected, and it's imperative that this process is acknowledged. Grief counseling, even for parents who did not lose their child, may be very helpful to these parents [2].

Caring for a child with a birth defect can negatively impact the physical and mental health of parents and caregivers. Many parents experience significant depression, fear and anxiety, which may have a devastating effect on the whole family if left untreated. These feelings are often suppressed due to embarrassment, shame or guilt. Mothers, who are often the primary caregivers of the children, may feel overwhelmed or that they can’t manage [2]. Fathers of children with birth defects will often say that they feel helpless—they believed they were supposed to protect their children, and they cannot protect them from birth defects. Well-intended comments from peers such as “you must be grateful your child is alive” or “at least it isn’t something worse” only worsens the feelings of guilt and may prevent parents from talking to others and seeking mental health services during a critical time. 

Parents of children with birth defects have the normal anxieties that come with raising children in addition to the many fears and anxieties that come with having a child with birth defects. They often feel ill-equipped to care for a child with special needs and wonder how they will manage emotionally, financially and logistically. Aside from the usual uncertainty that new parents face in the postnatal period, parents who have a baby in the newborn intensive care unit (NICU) or with medical interventions are now struggling to learn various nursing skills in addition to general parenting techniques. The mainstream parenting advice rarely applies to their child because there are few resources for parents who are dealing with children whose first day home is months after birth, who may have tubes or attachments to their tiny bodies, who suffer from sensory integration issues that none of the “normal” calming techniques will soothe, and who reach milestones at a disparate level compared to their peers. In fact, so many parents (particularly mothers) already blame themselves for “causing” their child’s condition *even in the absence of any medical evidence of causation*, that the additional feelings of inadequacy stemming from being overwhelmed with their child’s needs can push them deeply into a depression. Multiple studies support the notion that the worse a child’s symptoms are, the worse a parent’s psychosocial functioning deteriorates over time. Two studies with the same conclusion looked specifically at heart defects and spina bifida respectively [4,5], with congenital heart defects (CHD) being the most prevalent birth defect group today. 

### 2.3. Impact on Quality of Life

There are many factors that impact a family’s quality of life after a child is diagnosed with a birth defect, and these may be lessened or worsened based on the severity of the birth defect. If the birth defect requires medical intervention, hospital stays, surgeries and other procedures, the family’s quality of life is much more likely to be negatively affected. Things that may be impacted include finances, interacting with others, siblings and other children in the family, and marital relationships.

Many families suffer a financial burden when they have a child who has a birth defect due to a variety of factors. Employment can often be detrimentally impacted for families with children who have birth defects. Parents may face resistance at work as their employers do not understand why they are constantly going to the doctor, and appointments are rarely available outside of traditional work hours. Some parents may lose jobs, need to change jobs, or even decide that one parent needs to quit his or her job and stay at home with the child, which changes the family’s lifestyle drastically. Parents who have to leave employment may feel resentful, and the “working” parent may also feel resentful as he or she has to carry the entire financial burden of the family. In extreme cases, families may divorce or move to another state so they can afford their child’s medical care. 

There are also other factors that impact family finances such as traveling for care (which may include hotel stays, meals, travel cost itself, and time away from work), co-pays and co-insurance (which can amount to large sums of out-of-pocket costs), medical costs, and other healthcare-related costs such as special equipment, housing changes, and specialized childcare arrangements. In some cases, the financial burden on families gets so great that families must change residences and adjust their standard of living, which can cause stress for all involved. If the child needs regular physical, occupational, or speech therapy, this can create debilitating financial strain (even if insurance covers some costs) which can stigmatize the child who has a birth defect. Siblings of the child who has a birth defect may feel particularly resentful when the financial situation of the family changes drastically.

Interaction with those outside the family can be greatly affected by having a child with a birth defect. These issues may continue even once a child has been released to “regular” life. Parents are often unwilling or unable to participate in typical social activities. If the child is still dependent on medical assistance or is medically fragile, parents may be afraid to have their child around others. As a child grows, there may be noticeable differences between the child and his or her peers resulting from his or her birth defect. Even if there have been surgical corrections, the scars left behind can bring inquisitive questions from strangers. In the early years, the parents are able to act as a buffer for their child and help to explain their child's circumstances, but during the school years the child is left to defend himself or herself against teasing because of the differences. The social isolation and diminished self-esteem that can arise out of these situations can greatly impact the family’s quality of life. 

Many parents live with a sense of isolation, particularly if the birth defect their child has is rare and there is little support. This can cause significant anxiety in social settings and even lead distressed parents to further isolate themselves because they feel “different” from their peers, which may increase their depression or mental health anguish. Parents with children who receive multiple therapies or who are differently-abled as a result of their birth defect feel further separated from their peers as there is little in common in raising their children. Even routine activities such as a trip to the local park can be difficult since the physical differences between healthy children and those with birth defects can be emphasized when the children are side-by-side. 

Siblings and other children in the family are often greatly impacted by the diagnosis of a birth defect. Siblings may feel neglected, and those feelings may be justified, but feelings of neglect may result in behavioral issues and/or depression. Parents often feel guilty about not being able to spend as much time with siblings, but they often don’t have the time or the energy left to care for all of the children equally. Some siblings resent the child who has a birth defect, and that can be difficult for them emotionally.

Finally, parents of children with birth defects often experience significant strain on their marital relationship because of the stress they experience. The day-to-day interactions with children who have birth defects change the dynamics of spousal interactions, and as a result, this particular demographic is more likely to divorce than their non-affected counterparts. This is especially true if parents have limited support from family, friends, or professionals. Multiple studies acknowledge this phenomenon, with one particular study from the *Journal of Family Law* stating simply that “families with special needs children have higher divorce rates” [6]. Parents caring for a child with birth defects seldom have time and energy left for their relationship. Also, children with birth defects may have emotional or behavioral difficulties, making them even more time and energy-consuming. Given that this population is already at greater risk for divorce/separation, and that children with birth defects or special needs (even minor ones!) experience traumatic events more acutely than those without, [7] and that medical symptoms can worsen post-separation [6], these parents need extra support. 

### 2.4. Helping Families Cope—Healthcare Professionals

Healthcare professionals can play an important role in helping families cope with the challenges involved for children who have birth defects. Healthcare professionals should take extra care to educate families on what to expect when caring for their child and how to manage their child’s care. With the initial diagnosis, parents are often unable to take in information that may help them. Healthcare professionals should remember to reiterate what they have told the families over multiple appointments even though it may seem redundant, because parents are often so overwhelmed that they often “recall little from the initial consultation” [8]. Although specialist visits creates challenges for families, these necessary appointments also allow them to absorb the diagnosis and emotionally prepare for caring for their child. It would be extremely helpful to designate someone to follow up with parents to make sure they are able to cope post-discharge. This person should pay close attention to signs of distress, and have resources available to parents to help them in cases where it is needed.

### 2.5. Helping Families Cope—Support Groups

When parents feel connected to a strong support system, it is easier to navigate the daily challenges inherent with having a child with a birth defect. A review was done on peer support for children with chronic disabling conditions that looked at nine different studies and seventeen papers. The qualitative studies reviewed, in addition to some quantitative studies, showed that peer support had positive impact on psychological health and outcomes, but the authors concluded that more research is needed in this area [9].

When parents connect with other parents of children with birth defects, they develop a shared social identity which can provide a feeling of hope as parents see one another successfully coping and as they support one another through the process of raising their children [9]. One Canadian study demonstrated the importance of parental hope related to quality of life in spina bifida. It suggests that parental hope has a stronger impact on quality of life than physical problems [10]. Similar studies are needed for a variety of birth defects, not just focusing on performance and ability to participate in activities, but how religious and personal beliefs as well as parental attitudes affect overall feeling of well-being for children and their families. 

A 2008 study notes that women react in two ways after receiving a birth defect diagnosis: “monitoring” or “blunting” [8]. Those who monitor will seek out as much information as they can about their child’s diagnosis in order to be prepared for any outcomes, while those who blunt will avoid seeking information unless they need it to make a decision. The study also notes that not all women’s informational needs are met by their doctors. The single best action a physician can take upon the initial diagnosis is to provide the family with information of a support group for those with their child’s condition. This meets the needs of the “monitoring” parents as they will have one place to seek out information immediately, and the “blunting” individuals as they will know where to turn if they decide to reach out. 

In the case of a prenatal diagnosis, obstetric providers should provide the parents with condition-specific and reliable support group information, if available. Although parents are capable of completing online searches themselves, they can overlook avenues of support in the initial post-diagnosis turmoil, and one of the most frequent frustrations of parents is finding out years later that support groups existed that they did not have access to early on. In addition, Internet searches can lead to scary and misleading information about the child’s condition. Obstetric providers should be able to provide direction to the most appropriate support groups for that family.

Support groups are almost always started by families who have dealt with the same condition, and become an invaluable resource to parents throughout the course of their child’s life. These groups provide a central location to find information, a place to trade stories and offer a sense of community amid isolation. Siblings and other family members can find comfort in these communities as well, since their needs are often sidelined but their perspectives provide important contributions. Parents are able to ask questions and compare experiences, and “veteran” parents can ease the worries for them a little bit. This bonding process can greatly help medical practitioners as the stress level of their patients and their parents is reduced, and the information given to them is reiterated by their peers. Furthermore, support groups hold potential for mutually beneficial relationships with academia as families are often eager to provide insight to studies which may help epidemiologists find answers for their children. Support groups are an excellent source of empowerment for all family members and can help families dealing with the biopsychosocial ramifications of birth defects find their light in the darkness.

*When Joshua was diagnosed with a heart defect, we felt so alone—like no one understood what we were going through. We had never heard of congenital heart defects before he was diagnosed, and despite good medical care, we had a lot of questions. Finally, we connected with other parents with children who had congenital heart defects—people who had been through the same open-heart surgeries and procedures. Not only did we have questions answered and feel more relaxed caring for Joshua, we made life-long friends. Also, Joshua feels more comfortable around kids with the same scars and the same limitations.*

The quality of information and attitude of the diagnosing doctors and other professionals will impact the hope parents have for the future of their baby. Connecting families to support groups and parent-to-parent networks can also positively impact parent attitude and feeling of belonging as they can relate with others [9]. These groups can be both local and online. Sometimes distance makes participation in local groups difficult but Internet and phone support helps breach this barrier. Support personnel should be careful to welcome new families so they do not feel unintentionally left out or overwhelmed at events. Many families make great efforts to attend support activities and often drive long distances or take off of work to seek out support, and this can be intimidating. It’s important to remember that the friendliness and understanding of staff and support groups has the potential to influence parents in a positive manner, generating more interest and interaction with them and others in the future.

*While still in the hospital after receiving the diagnosis of our son’s birth defect, we were not told of any other parents in the immediate area with whom we could connect. After his birth, the support organization in the area came to clinics and took our information, but we only received a couple of mailings and only one contacted us. The one activity I did attend was an hour’s drive and required much effort. The people helping at booths were nice, but not one person came to welcome us when we were obviously new and overwhelmed. For some this may not be a problem, but for others like me, a new social situation can be terrifying, especially when you are still trying to deal with the implications of your child’s defect. It would have been nice to connect with people closer to home and to feel welcomed.*

## 3. Communication

Communication with parents of children who have birth defects is vitally important. Healthcare professionals need to take every opportunity to leave the lines of communication open with parents and to help them along their journey from the initial diagnosis to essential educational information and finally to the future needs and issues for that child.

### Communication of Diagnosis

Communication of a diagnosis of a birth defect in a baby or child is certainly very difficult. Different parents will react differently—some taking it fairly well, some falling apart completely, and everywhere in between [2]. While there are many unknowns regarding birth defects, especially prenatally, medical professionals need to be as clear as possible with them. It is an overwhelming time and vagueness can prove frustrating. Whenever possible, a diagnosis should be communicated in clear and simple terms, but also with compassion. 

It is of utmost importance that parents are given access to the most current and relevant information pertaining to their baby’s birth defect. It is also important to provide information they can understand based on culture and language. Any written materials should be written in terms that are not heavily laden with medical jargon they may not understand [11].

*When we were told our son would have spina bifida, we were given information that was outdated, poorly copied and folded. I read very little from those fliers because I felt that the information would not be correct or current. As a mom, this caused me to feel like I did not have adequate information to help me care for my son, and therefore made me have more anxiety about his care. This could have been avoided simply by finding more updated* *and relevant information that also looked current and professional.*

A diagram or picture often helps. In today’s world, there are also many apps or computer programs that can be shown to parents helping them understand. If the parents want more information, know where they can find *trustworthy and reliable* information knowing that they are going to go search the Web, which can be a very scary place in regards to birth defects and diseases.

Excellent information on birth defects is available to healthcare professionals and can be extremely helpful when shared with families. It is also helpful for healthcare professionals to participate in continuing education in order to obtain skills and tools to communicate effectively with a variety of patients and to be able to provide the most current medical information possible. 

When communicating a diagnosis, it is also critical to the health of the baby or child that the parents receive information about necessary care and access to needed resources. The diagnosing doctor should ensure that the parents are immediately connected to the appropriate clinics and specialists, and that communication with the parents is maintained to ensure they understand what is needed in the way of medical care and other resources. Parents need to know the plan of action and what to expect, not just immediately, but in the future as well. 

In addition, parents need access to services for their baby or child so that he or she can receive the best possible care. Medical services are essential to families, but they often need more than just medical services. Physical, occupational, speech, vision or other therapy is often needed and should begin as soon as possible once the baby is healthy enough and ready to begin, and depending on the stage of development and needs. Because having a child with a birth defect can cause a great deal of strain on individuals, marriages and families, parents may need to be given information about mental health services and help connecting with those services, if needed. This can be done by primary care providers, clinics, therapists and support groups. Recreational and adaptive sports can also provide great opportunities for support and connecting to other families. Support groups can also be an important part of the healing process, and healthcare professionals should inform parents about any support groups for their child’s defect in their area.

Many factors can contribute to the successful integration of families into actively participating in available services, and it is important to help families overcome barriers to access. Barriers might include accessibility of provider offices, such as wheelchair accessibility, distance to services and transportation availability. Whenever possible, satellite or mobile clinics can help to reach a greater amount of families for basic and follow-up care. Other barriers include the provider’s knowledge about the birth defect and the provider’s ability to communicate with the patient and/or parents. Language, culture, ethnicity, income and education can all affect access to services. These barriers may even affect understanding of why certain services or procedures may be needed. The knowledge and friendliness of the service’s staff can also impact parents in either a positive or negative manner and can be a factor in continuing care. In addition, lack of insurance may impede access to needed or additional services such as mental health care, needed supplies or adaptive equipment [12]. 

## 4. Parents as Prevention Partners

New parents of children with birth defects often gravitate toward two questions:

1. Why did this happen to my child?
2. How do we prevent this from happening again with another child?

Accordingly, prevention research, education and outreach have special meaning to parents asking these difficult questions.

There are three levels of prevention research pertaining to birth defects. Primary prevention is designed for action to be taken before pregnancy occurs in order to reduce the occurrence of birth defects. The fortification of foods and grains with folic acid along with educating women about taking a multivitamin with folic acid is an example of primary prevention to reduce the risk of having a baby with a neural tube defect. Secondary prevention in birth defect research looks at detecting a disease when it is asymptomatic in order to prevent complications or even early death. An example of this is the newborn screening for critical congenital heart defects using pulse oximetry. Tertiary prevention aims to minimize the impact of the birth defect on the child’s system as a whole. Parents can be powerful partners at each of these levels by advocating for research, disseminating information, pointing out neglected issues, and providing support for other parents.

### 4.1. Advocating for Research

Parents struggling to answer the “why” question find great hope in basic research and epidemiological studies. Even if such research has no immediate direct impact to their child, parents are often tireless and strong advocates for funding and attention in an effort to prevent future defects in others. The challenges lie in connecting parents and funding for these efforts, along with the changing needs of the upcoming generations.

*My daughter Ashley was born with gastroschisis and microtia/atresia. The gastroschisis had to be repaired at birth in order for her to survive, and the microtia/atresia left her hearing impaired. When she was born eight years ago, doctors said that it was just a “fluke” and very rare. Since then, the prevalence of gastroschisis has more than quadrupled; rates are still on the rise and nobody knows why. Research funding is being cut, and this impacts our family and future generations. I have spent her entire life advocating for more research so that other families have answers and so we can stop this birth defect from increasing even more. My daughter is healthy now, with only minor health repercussions and a few scars as a reminder of her struggles and surgeries, but I want to prevent other children from having to endure the same experiences. She has become quite an advocate herself, and she will tell people what she was born with while explaining that “she was just made this way nobody knows why.” I’m confident that one day Ashley will be able to tell people the “why” of her birth defects.*

As members of the Parent Advisory Group for the National Birth Defects Prevention Network (NBDPN), a group of parents has been able to lobby legislative leaders for increased funding for surveillance and research to improve our understanding of what environmental factors can be modified to reduce the risk of birth defects and support prevention efforts on the population level. Armed with the knowledge of prevalence and epidemiologic data, our Parent Advisory Group can successfully educate state and federal leaders for funding to move toward change. It is through this collective partnership of parents, researchers, physicians and other key stakeholders change can occur. 

Parents would like to see further research into effective means of preventing birth defects, for new treatment options for their children and for prevention of secondary issues that often come with birth defects. The National Birth Defect Network Prevention Study (NBDPS) [13] has been looking to answer some of these questions. Since 1997, the Centers for Disease Control and Prevention (CDC) and nine states have conducted a population-based case-control study investigating maternal and paternal exposures and whether certain exposures increase the risk for specific birth defects. As parents we recognize and value the importance of the state-wide surveillance system as a means to identify clusters or patterns in prevalence. Accordingly the information we glean from the surveillance system will direct future primary prevention efforts and education campaigns. But all these efforts take proper funding.

Parents can also advocate for research by participating in studies and recruiting other parents to participate.

*As a parent I was asked to participate in the National Birth Defect Prevention Network Study. As a participant, I was asked personal questions about many aspects of my pre-conception health and pregnancy. This caused me to gain awareness about my personal habits when considering subsequent pregnancies. I found that I was much more aware of potential teratogens including caffeine, smoking and other environmental dangers that may adversely affect my pregnancies. As this study identifies more factors that may increase the risk of birth defects, we will begin to find answers to the why question.*

### 4.2. Disseminating Information

When the “why” questions in regards to birth defects have been answered, parents can be key figures in disseminating this information to target audiences at all three prevention levels.

After we know why birth defects occur, risk factors can be identified. Once risk factors are identified, primary prevention must occur—the public must be informed and prospective parents must take action in order for women to reduce their risk of a having a child with a birth defect. In other words, just knowing what leads to birth defects is not enough. We must then translate this knowledge into action by identifying risk factors and informing the public about them in order to prevent birth defects. 

One of the challenges for contemporary primary prevention information as a result of research is the difficulty in disseminating the information to the targeted population. As many new women enter childbearing age each year, the information needs to be continually disseminated. In order to enact a change in behavior, information dissemination has to be targeted directly to the specific population of women and is a very time-intensive process. For example, current prevention research indicates that there are successful methods which can be implemented to facilitate optimal health for newborns. Smoking cessation and no alcohol prior to becoming pregnant should be top on the list of priorities to ensure the health of a baby. Unfortunately, many women do not understand or choose to ignore these known teratogens. Another issue is that women do not understand that by the time they recognize their pregnancy (e.g., eight weeks after their last menstrual period) many of the organs have developed (normally or abnormally), so care must be taken *before* pregnancy, which becomes even more difficult in unplanned pregnancies.

Many examples of efforts to educate women about primary prevention and birth defects in general have been conducted. Basic and epidemiological research identified the use of folic acid before pregnancy as a key factor for the prevention of neural tube defects [14]. However, only 20% of women surveyed were aware that folic acid prevents birth defects and less than 40% actually take a daily multvitamin with folic acid [15]. In an effort to raise awareness of birth defects, the Parent Advisory Group of the NBDPN developed an educational campaign which was launched January 1, 2013. As part of the educational campaign, a public service announcement was created and disseminated worldwide [16].

Communication of current research to prospective parents by clinicians and researchers can be done in an appropriate and sensitive way. When it comes down to prevention, efforts need to be marketed aggressively. We need public service announcements that people will watch and relate to. When constructing materials and data charts, it is important for researchers to identify key points and clearly state them so that they are on a 5th grade reading level and are understandable by people from different cultural backgrounds. By maintaining current knowledge of social networking and changing technological environments, researchers should be sensitive to the different means of communicating with the targeted population for prevention messages. Current trends and social networking sites can be used as ways to disseminate information about prevention efforts concerning birth defects.

### 4.3. Pointing out Neglected Issues

It is crucial that a child with a birth defect has supportive environments available for care. One often neglected issue involves co-morbidities related to secondary and tertiary prevention. Often times a child with a birth defect has multiple co-occurring issues to be addressed. A multidisciplinary approach can provide the necessary support to address all issues without inconveniencing families excessively. Families are inconvenienced already as they navigate the system and have many new technologies to learn, as is the case with spina bifida and CHD. Families are often overtaxed with the expenses and time that is required to travel to and from the hospital. Providing the convenience of a clinic day allows them to make one trip and get all their appointments finished in one day.

Children with birth defects are at risk for co-morbidities. If children are seen in a team setting it is easier for the team of physicians to communicate together about best practice for a particular child and their family.

*The craniofacial team has been very helpful in facilitating a full service support for Jacob and our family. Besides actually repairing Jacob’s cleft lip and palate, they have helped us to identify and subsequently seek treatment for feeding issues, speech support, audiological interventions, orthodontic care, sleep and pulmonology issues, psychological services, gastrointestinal issues and provide financial direction in order to pay for all the treatments Jacob requires. This has been such a support for our family.*

Tremendous technological advances have been made in surgical procedures to correct and ameliorate birth defects; however, supportive technologies are often neglected. Parents are left to create their own adaptations for support. If these kinds of technologies were systematically researched and developed, more supportive products would be available.

*Jacob was born with a cleft lip and palate. We had to have multiple surgeries to correct his lip and palate. We were required to constantly monitor him so he would not put anything in his mouth. The restraints provided after surgery were not effective and continually slipped off of Jacob’s arms or poked him in the face. We were forced to develop an elaborate diaper pinning system that worked more efficiently in our situation. In addition, his nasal stents did not stay in place and this ultimately had an impact on his nasal development.*

This example illustrates the need not only for research on birth defects *per se*, but for technology and information on how to care for a child post-surgery to enhance recovery.

### 4.4. Consistency of Care across Settings

Families should receive timely information about general procedural care for their child.

*Our three year old son with cleft lip and palate was initially seen at a craniofacial clinic in the west that offered a new procedure designed to prevent subsequent jaw surgery. As we moved across the country we discovered that the clinic at the new location did not offer this particular procedure. Because of the clinic’s differing views on best practice, our son was subsequently required to undergo the more invasive jaw surgery.*

For parents, the lack of consistent information and procedures is a common issue. It can be very frustrating when trying to choose what the parent believes to be the best care for their child. Many families that live in rural areas do not have access to specialty clinics. Sometimes these children are unable to receive treatment that is optimal. With changing technological advancements, these new approaches to surgical intervention need to be shared with parents in a way that help them make educated, informed decisions about their child’s care. Tertiary preventative methods can be helpful to families and patients, if the families can access them. 

## 5. Transition to Adult Care for Children with Birth Defects

Being a parent of a child with a birth defect can be very difficult, particularly when it comes to helping that child learn to take care of his own health as an adult. When parents have a child with a birth defect, they often have taken extra measures to keep their child healthy. Many of these parents have seen more than one medical specialist with their child, worked tirelessly to educate themselves about their child’s condition, protected their child from germs, kept track of medications, managed the medical system, etc. So when it comes to helping a child transition to adult care, parents can feel scared and unsure about how to move forward. It is often hard to let go and trust that a child has what it takes to care for himself or herself, yet it is necessary.

### 5.1. Start Early

While many parents don’t start thinking about their child transitioning to adult care until the teenage years, transitioning needs to start before then. If children feel capable of making good decisions and have the necessary information, they are more likely to be successful in taking charge of their health when they are out on their own. Children who don’t understand their condition, why they are taking the medications, and why they need to see a doctor or specialist regularly, are less likely to transition well. Some children actually stop taking important medication as adults because they didn’t understand what the medication was for, and they didn’t like the way it made them feel. Likewise, children might not know what activities are okay for them to engage in if they really don’t understand their limitations. On the other hand, kids who do understand their condition often report a better than average quality of life—they feel in control and empowered and are often more successful in taking care of themselves [17].

### 5.2. Understanding Their Condition

If possible, children should start to learn about their condition early in an age-appropriate way. Hospital social workers can be very helpful with this process. Children should be allowed to ask questions as soon as they can and learn about their birth defect, what medications they are taking and what they are for, and what activities are not safe for them and why.

*Joshua was born with a congenital heart defect called Hypoplastic Left Heart Syndrome and had three open-heart surgeries before the age of three. The name of his heart defect, alone, is way too much for even most adults to say or understand, so when Joshua was about four-years-old, we explained to him that he had a special heart that needed special care. At five years of age, he learned that he was born with Hypoplastic Left Heart Syndrome, what he now calls “half-a-heart,” or sometimes a “right heart.” We let him ask his health care providers questions and they, in turn, talk to him about his heart defect, his scar, medications he needs, and his activities.*

When explaining a child’s condition, it is very important that the child doesn’t feel bad about his condition. Many birth defects actually may make kids stronger and more resilient than other children because they learn how to overcome adversity early [18,19,20].

*Joshua feels that he is braver than other kids because he made it through three open-heart surgeries for the staged repairs of his Hypoplastic Left Heart Syndrome. This belief in himself has given him good self-esteem despite his limitations, and he has a can-do attitude.*

If your child has limitations, focus on what the child ***can*** do versus what he or she ***can’t*** do. A child’s attitude will also help or hinder the child taking care of his or her own healthcare needs when the time comes. A child who believes she is strong and smart will be more empowered to care for herself.

### 5.3. Allowing Questions

As children get older, they often have questions about their condition. Parents may have many of the answers, but it is often helpful to allow the child to ask her doctor or other medical professional these questions. Some children simply need their condition explained by someone who is used to talking to children about that particular condition. Often children will want to know what their limitations are, if any, and what sports and activities they can and can’t engage in. Sometimes they will have questions parents haven’t even thought of. Also, sometimes hearing the “why” from a doctor or healthcare professional about the inability to participate in desired activities is much easier for the child to accept than the “no” from a parent.

A simple way to allow a child to ask questions about his condition is to turn to the child during the visit to the doctor and ask him whether he has any questions for the doctor. This makes children feel important and helps them learn that they need to understand their condition. It also give them a feeling of some control over their condition, which is very important to their self-esteem.

### 5.4. Locate Specialists and Resources

It can be very helpful if parents can identify resources for their child to access when they are out on their own before that time comes. There are many resources available for children who have birth defects as they become adults. Most State Departments of Health will have that information available. The National Birth Defects Prevention Network has important links available on its website [16]. 

Also, if parents know their child will need to see a specialist, parents can help that child find the specialist and make a visit before the child leaves home. For example, adults with CHD need to visit an adult cardiologist who specializes in congenital heart defects. It is important to locate one during the teen years so that there is not a gap in the child’s care. A listing of adult congenital heart specialists can be found online [17]. 

### 5.5. Empower Your Child

Parents with children who have birth defects sometimes over-protect their children. This over-protection is out of a desire to keep their children safe and healthy. However, over-protection can make a child feel that he is incapable of making good decisions and that he does not know how to do things on his own. This can have unintended negative consequences as children become adults and need to care for themselves.

Healthcare professionals should encourage parents to empower their children by letting them make choices, and sometimes making mistakes, within reason. We all learn to walk by falling down many times. Likewise, children learn how to make good decisions by making bad ones sometimes and suffering the consequences. Parents need to understand that, as much as possible, children with birth defects should be made to feel “normal”—like any other kid. Healthcare professionals can clearly explain what the child can do and help the parents feel as positively as possible about their child’s abilities.

### 5.6. Research Areas

Research is needed throughout the life span (*i.e.*, before conception for primary prevention and thereafter for efforts to improve the health of children with birth defects). Table 1 depicts the themes presented and the necessary tasks to help improve the lives of children born with birth defects and their families. To gain a better understanding of the transition process in the United States, it would be beneficial to understand how many children who need to see a specialist as adults are lost to care and by what age. It would also be helpful to assess a child’s level of understanding of his or her condition when the child reaches adulthood. Finally, a study could be done on parental behavior that determines what the most appropriate method would be to create a high perceived quality of life for children with birth defects as they reach adulthood. 

**Table 1:** Common themes among parents of children with different types of birth defects.

THEME	ISSUE	(FUTURE) NEED
*Communicating, Coping and Adapting*	Diagnosis	Survey parents on preferred communication techniques; train physicians on methods of effective communication to parents based on results
		Provide information to parents during prenatal or postnatal diagnosis with compassion and include referral to appropriate services or support groups
	Support	Increased collaboration between medical providers and patient support/advocacy groups
		Encourage practitioners to refer patients to support groups as part of standard procedure
	Information	Research best methods for information dissemination to parents
		Empower parent and advocacy groups to contribute to research
		Means to connect parents, patients, and healthcare professionals for more effective advocacy
	Resources	Develop reliable resources for parents, particularly web-based resources for instant access
		Increased access to support devices, technologies, and therapies needed
		Creation of a national 800# for parents to call in for information, support, and referrals
*Quality of Care*	Clinician Knowledge	Ensure open communication amongst clinicians regarding the most up-to-date research studies
		Creation of multi-disciplinary clinics
	Consistency of care	Find a way to standardize quality of care nationwide
		Consistent follow up with affected families
	Understand co-morbidities	Additional research on occurrence and prevention
*Quality of Life*	Prenatal	Increased understanding of ways to improve parental coping mechanisms
	Postnatal	Studies on prevalence and treatment of sensory disorders and behavioral issues
		Develop effective therapies to help diminish these behaviors
	Financial	Determine comprehensive cost of care burdens on families including non-medical and mental health costs
	Social	Assess social issues and stigmas associated with birth defects and how to minimize negative impact
	Parenting	Assess benefits of peer-to-peer support for families
		Encourage social/emotional/psychological support of families in conjunction with social workers or other support staff
	Resiliency	Determine factors that improve resiliency of parents and the child
		Start dialogues within the community that normalizes the needs and experiences of children with birth defects
*Transition*	Parent Training	More resources available on raising a child with birth defects post-early childhood
		Provide access to care trainings through local special needs or disability agencies, hospitals, or community care providers
	Physician Training	Increased education for specialists on appropriate lifelong care for children with birth defects
		Studies on loss of care of children as they reach adulthood
	Empower Children	Educate parents, caregivers, and clinicians on the importance of open discussion while allowing the child to ask questions and have input on decisions
		Study of parental behavior and its impact on children seeking appropriate care as adults
		Ensure that insurance companies will pay for therapies and other needed intervention intended to maximize child's potential

## 6. Conclusions

Birth defects are common, costly and critical. They impact the quality of life of the whole family, often in negative ways. For the vast majority of birth defects we do not know why they occur. More research is needed. We want to increase the likelihood that all babies are born without birth defects and prevention of secondary issues and co-morbidities. For those that we cannot prevent, we urge you to help us make a difference in their lives. This takes a collective effort to educate the general public, policy makers, and all health care providers. It takes work on communication by healthcare professionals so that parents understand their child’s birth defect, necessary medical care, how to find current and relevant information. Parents need to have access to services, including support groups that can help them care for their child, in addition to benefitting the entire family. It also takes a commitment by Congressional leaders at the federal level as well as the state level to support the necessary research. We hope this article provides a beneficial insight into our experiences. 
